# Supplementary material for: Causes and consequences of sex-chromosome turnovers in Diptera
Source: Evol Lett. 2026 Mar 12;10(3):253–68. doi: 10.1093/evlett/qrag003 (PMC13231274; doi:10.1093/evlett/qrag003)
Supplement: qrag003_Supplemental_Files [file qrag003_supplemental_files.zip › Supplementary_Tables 4.zip!/Supplementary_Tables 4/Table_S1_List_species_citations.pdf]

Supplementary Table 1. List of analysed genomes and corresponding citations.

Species	Family	D. mel genes	Chr number	Genome size	Common-family name	X-linked element(s)	Citation	Genome
Acrocera orbiculus	Acroceridae	6836	4+X	221 Mb	small-headed flies	?	(Crowley *et al.*, 2024b)	GCA_947359355.1
Pegoplata infirma	Anthomyiidae	8990	5+X+Y	1.4 Gb	root-maggot flies	F	(Falk *et al.*, 2024b)	GCA_963921195.1
Dysmachus trigonus	Asilidae	7816	5+X+Y	450.3 Mb	robber flies	F	(Sivell et al., 2024b)	GCA_949715965.1
Eutolmus rufibarbis	Asilidae	7792	6+X	285.9 Mb	robber flies	F	(Nash et al., 2024b)	GCA_963920795.1
Machimus atricapillus	Asilidae	7770	4+X+Y	268.6 Mb	robber flies	E,F	(Thomas *et al.*, 2023)	GCA_933228815.1
Machimus rusticus	Asilidae	7732	6+X+Y	279.7 Mb	robber flies	F	(Mitchell et al., 2024a)	GCA_951509405.1
Tolmerus cingulatus	Asilidae	7759	4+X+Y	280 Mb	robber flies	F	(Mitchell et al., 2024b)	GCA_959613345.1
Bibio marci	Bibionidae	6383	5+X	340 Mb	march flies	F	(Sivell et al., 2021a)	GCA_910594885.2
Dilophus febrilis	Bibionidae	6266	5+X	305.6 Mb	march flies	F	(Falk et al., 2025b)	GCA_958336335.1
Anthrax anthrax	Bombyliidae	7086	7+X+Y	334.6 Mb	bee flies	C,F		GCA_963971155.1
Bombylius discolor	Bombyliidae	7091	5+X	279.8 Mb	bee flies	C,F	(Broad et al., 2022)	GCA_939192795.1
Bombylius major	Bombyliidae	7073	5+X+Y	304.3 Mb	bee flies	C,F	(Lawniczak et al., 2023)	GCA_932526495.1
Villa cingulata	Bombyliidae	7033	8+X+Y	412.6 Mb	bee flies	C,F	(Taylor et al., 2023)	GCA_951394055.1
Bellardia pandia	Calliphoridae	8896	5+X	617 Mb	blow flies	F	(Falk et al., 2021a)	GCA_916048285.2
Calliphora vomitoria	Calliphoridae	9000	5+X	707.6 Mb	blow flies	F	(Sivell et al., 2023d)	GCA_942486065.2
Protocalliphora azurea	Calliphoridae	8918	5+X+Y	874.2 Mb	blow flies	F	(Falk et al., 2023b)	GCA_932274085.1
Stomorhina lunata	Calliphoridae	8928	5+X	728.1 Mb	blow flies	F	(Mitchell et al., 2023b)	GCA_933228675.1
Clusia tigrina	Clusiidae	8843	3+X+Y	1.2 Gb	druid flies	D	(Crowley et al., 2023b)	GCA_920105625.2
Coelopa pilipes	Coelopidae	8460	5+X+Y	263 Mb	kelp flies	F	(Butlin et al., 2024)	GCA_947389925.1
Conops quadrifasciatus	Conopidae	7778	5+X+Y	210.4 Mb	thick-headed flies	F	(Falk et al., 2024c)	GCA_949752815.1
Myopa tessellatipennis	Conopidae	8050	3+X	249.3 Mb	thick-headed flies	unknown X	(Ashworth et al., 2023)	GCA_943737955.2
Myopa testacea	Conopidae	8042	3+X+Y	243.3 Mb	thick-headed flies	D,F	(Falk et al., 2024i)	GCA_949629155.1
Physocephala rufipes	Conopidae	7567	5	243.3 Mb	thick-headed flies	unknown X		GCA_963966595.1
Sicus ferrugineus	Conopidae	7851	5+X+Y	311.9 Mb	thick-headed flies	A,F	(University of Oxford and	GCA_922984085.1
Thecophora atra	Conopidae	8187	3+X+Y	354.2 Mb	thick-headed flies	B	(Mitchell et al., 2023a)	GCA_937620795.1
Anopheles albimanus	Culicidae	6219	4+X	170.2 Mb	mosquitoes	A,F	(Lukyanchikova et al., 202	GCA_015501965.1
Anopheles aquasalis	Culicidae	6350	2+X+Y	176.6 Mb	mosquitoes	A,F	(Alencar et al., 2023)	GCF_943734665.1
Anopheles arabiensis	Culicidae	6686	2+X	256.8 Mb	mosquitoes	A,F	(Zamyatin et al., 2021)	GCF_016920715.1
Anopheles atroparvus	Culicidae	6669	4+X	220.1 Mb	mosquitoes	A,F	(Lukyanchikova et al., 202	GCA_015501955.1
Anopheles bellator	Culicidae	6581	2+X	169.6 Mb	mosquitoes	A,F		GCF_943735745.2
Anopheles coluzzii	Culicidae	6603	4+X	245.4 Mb	mosquitoes	A,F	(Zamyatin et al., 2021)	GCA_016508615.1
Anopheles coustani	Culicidae	6769	2+X	270 Mb	mosquitoes	A,F	(Bouafou et al., 2024)	GCF_943734705.1
Anopheles cruzii	Culicidae	6597	2+X	84.1 Mb	mosquitoes	A,F		GCF_943734635.1
Anopheles darlingi	Culicidae	6126	2+X	181.6 Mb	mosquitoes	A,F	(Gendrin et al., 2025)	GCF_943734745.1
Anopheles funestus	Culicidae	6845	2+X	250.7 Mb	mosquitoes	A,F	(Ayala et al., 2023)	GCF_943734845.2
Anopheles gambiae	Culicidae	6655	2+X	264.5 Mb	mosquitoes	A,F	(Habtewold et al., 2023)	GCA_943734735.2
Anopheles longipalpis	Culicidae	7014	4+X	347.1 Mb	mosquitoes	A,F	(Small et al., 2020)	GCA_016170015.1
Anopheles maculipalpis	Culicidae	6796	2+X	224.1 Mb	mosquitoes	A,F	(Rahola et al., 2024)	GCF_943734695.1
Anopheles marshallii	Culicidae	6833	2+X	225.7 Mb	mosquitoes	A,F	(Makanga et al., 2024)	GCF_943734725.1
Anopheles merus	Culicidae	6726	4+X	294.4 Mb	mosquitoes	A,F	(Lukyanchikova et al., 202	GCF_017562075.2
Anopheles moucheti	Culicidae	6809	2+X	271.3 Mb	mosquitoes	A,F	(Nsango et al., 2023)	GCF_943734755.1
Anopheles nili	Culicidae	6538	2+X	195.2 Mb	mosquitoes	A,F	(Nsango et al., 2024)	GCF_943737925.1
Anopheles parensis	Culicidae	6934	4+X	279 Mb	mosquitoes	A,F	(Small et al., 2020)	GCA_016254315.1
Anopheles rivulorum	Culicidae	6737	2+X	321.5 Mb	mosquitoes	A	(Small et al., 2020)	GCA_018908115.1
Anopheles stephensi	Culicidae	6947	4+X	307.5 Mb	mosquitoes	A,F	(Srinivasan et al., 2022)	GCA_023078585.1
Anopheles vaneedeni	Culicidae	6840	1+X	308.7 Mb	mosquitoes	A,F	(Small et al., 2020)	GCA_016170025.1
Anopheles ziemanni	Culicidae	6766	2+X	270 Mb	mosquitoes	A,F		GCF_943734765.1
Teleopsis dalmanni	Diopsidae	8732	2+X	457.8 Mb	stalk-eyed flies	B	(Reinhardt et al., 2023)	GCA_002237135.5
Dolichopus griseipennis	Dolichopodidae	7061	3+X+Y	944 Mb	long-legged flies	F	(Ashworth et al., 2025)	GCA_963082915.1
Poecilobothrus nobilitatus	Dolichopodidae	7172	5+X+Y	897.5 Mb	long-legged flies	F	(Mitchell et al., 2024d)	GCA_947095535.1
Drosophila albomicans	Drosophilidae	10158	4+X	167.5 Mb	vinegar flies	A,C,D*	(Mai et al., 2020)	GCF_009650485.2
Drosophila ananassae	Drosophilidae	11725	5+XL+XR	213.9 Mb	vinegar flies	A	(Leung et al., 2023)	GCA_030555595.1
Drosophila athabasca	Drosophilidae	11232	3+X	192.7 Mb	vinegar flies	A,C,D*	(Bracewell et al., 2019)	GCA_008121215.1
Drosophila busckii	Drosophilidae	10023	5+X	118.5 Mb	vinegar flies	A,F**	(Renschler et al., 2019)	GCF_011750605.1
Drosophila elegans	Drosophilidae	12578	5+X	294.7 Mb	vinegar flies	A	(Massey et al., 2020)	GCA_011057505.1
Drosophila innubila	Drosophilidae	10154	5+X	166.3 Mb	vinegar flies	A	(Hill et al., 2019)	GCA_004354385.2
Drosophila kepulauana	Drosophilidae	10210	4+X	163.8 Mb	vinegar flies	A	(Mai et al., 2020)	GCA_023558455.1
Drosophila lowei	Drosophilidae	10939	4+X	184.3 Mb	vinegar flies	A,D	(Bracewell et al., 2019)	GCA_008121275.1
Drosophila mauritiana	Drosophilidae	12764	5+X	152.3 Mb	vinegar flies	A	(Chakraborty et al., 2021)	GCF_004382145.1
Drosophila melanogaster	Drosophilidae	12859	5+X+Y	143.7 Mb	vinegar flies	A	(Hoskins et al., 2015)	GCF_000001215.4
Drosophila miranda	Drosophilidae	11149	4+XL+XR	287.1 Mb	vinegar flies	A,C,D*	(Mahajan et al., 2018)	GCF_003369915.1
Drosophila nasuta	Drosophilidae	10272	4+X	171.8 Mb	vinegar flies	A	(Mai et al., 2020)	GCF_023558535.2
Drosophila pallidifrons	Drosophilidae	10303	4+X	164.7 Mb	vinegar flies	A	(Mai et al., 2020)	GCA_023558445.1
Drosophila pseudoobscura pseudoobs	Drosophilidae	11100	4+X	163.3 Mb	vinegar flies	A,D	(Richards et al., 2005)	GCF_009870125.1
Drosophila sechellia	Drosophilidae	12733	5+X	153.1 Mb	vinegar flies	A	(Chakraborty et al., 2021)	GCF_004382195.2
Drosophila subpulchrella	Drosophilidae	12363	5+X	265.2 Mb	vinegar flies	A	(Durkin et al., 2021)	GCF_014743375.2
Drosophila sulfurigaster albostrigata	Drosophilidae	10159	4+X	168.3 Mb	vinegar flies	A	(Wei et al., 2022)	GCF_023558435.1
Drosophila sulfurigaster bilimbata	Drosophilidae	10333	4+X	164.6 Mb	vinegar flies	A	(Wei et al., 2022)	GCA_023558465.2
Drosophila sulfurigaster sulfurigaster	Drosophilidae	10133	4+X	165.9 Mb	vinegar flies	A	(Wei et al., 2022)	GCA_023558475.1
Drosophila triauraria	Drosophilidae	11279	5+X	195.3 Mb	vinegar flies	A	(Torosin et al., 2020)	GCA_014170255.2
Drosophila virilis	Drosophilidae	10362	5+X	169.8 Mb	vinegar flies	A	(Hemmer et al., 2020)	GCA_007989325.2
Dryomyza anilis	Dryomyzidae	9040	5+X+Y	656.6 Mb		F	(Sivell et al., 2024c)	GCA_951804985.1
Empis livida	Empididae	7361	5+X	291.2 Mb	dragger flies	F	(Falk et al., 2024h)	GCA_963932195.1
Rhamphomyia laevipes	Empididae	7348	5+X+Y	394.5 Mb	dragger flies	F	(Falk et al., 2024m)	GCA_963920725.1

Suillia variegata	Heleomyzidae	8683	5+X+Y	264 Mb	sun flies	F	(Falk et al., 2023h)	GCA_949127995.1
Ornithomya chloropus	Hippoboscidae	7855	3+X+Y	193.2 Mb	louse flies	B, C	(Wawman et al., 2025a)	GCA_963971445.1
Ornithomya fringillina	Hippoboscidae	7827	5+X	172.8 Mb	louse flies	B	(Wawman et al., 2025b)	GCA_963978525.1
Hybos culiciformis	Hybotidae	7234	3+X+Y	342.6 Mb	dance flies	F		GCA_964007475.1
Calliopum simillimum	Lauxaniidae	8987	5+X	690.5 Mb	Lauxaniid flies	F	(Falk et al., 2024n)	GCA_951812925.1
Meiosimyza decipiens	Lauxaniidae	8970	4+X+Y	436.4 Mb	Lauxaniid flies	F	(McCulloch et al., 2025a)	GCA_963680825.1
Eudasyphora cyanicolor	Muscidae	9012	5	1.5 Gb	house flies	unknown X	(Falk et al., 2025a)	GCA_963930755.1
Haematobia irritans	Muscidae	7742	scaffolded	1.1 Gb	house flies	scaffolded	(Konganti et al., 2018)	GCA_003123925.1
Hydrotaea cyrtoneurina	Muscidae	8940	6	575.2 Mb	house flies	unknown	(Falk et al., 2024e)	GCA_958296145.1
Hydrotaea diabolus	Muscidae	9021	5+B1+B2	1.1 Gb	house flies	unknown	(Falk et al., 2024f)	GCA_963513945.1
Musca domestica	Muscidae	9031	scaffolded	750.4 Mb	house flies	scaffolded	(Meisel et al., 2023)	GCA_000371365.1
Muscina levida	Muscidae	8965	5	805.2 Mb	house flies	unknown	(Falk et al., 2024g)	GCA_964034835.1
Phaonia tiefii	Muscidae	9019	5	1.5 Gb	house flies	unknown	(Nash et al., 2024a)	GCA_963931995.1
Philornis downsi	Muscidae	9001	scaffolded	954.8 Mb	house flies	scaffolded	(Romine et al., 2022)	GCA_019455685.1
Polietes domitor	Muscidae	8988	5+X	1 Gb	house flies	?	(Falk et al., 2024l)	GCA_947397865.1
Stomoxys calcitrans	Muscidae	8989	5	1.1 Gb	house flies	unknown	(Sims et al., 2025)	GCF_963082655.1
Toxonevra muliebris	Pallopteridae	9079	4+X+Y	491.4 Mb	flutter flies	F	(Barclay et al., 2025)	GCA_963691655.1
Nephrocerus scutellatus	Pipunculidae	8408	5+X	613.4 Mb	flutter flies	F	(Falk et al., 2024p)	GCA_947095585.1
Pollenia amentaria	Polleniidae	8953	5+X	1.3 Gb	cluster flies	F	(Falk et al., 2023d)	GCA_943735925.1
Pollenia angustigena	Polleniidae	8989	5+X	1.4 Gb	cluster flies	F	(Falk et al., 2023j)	GCA_930367215.1
Ptychoptera albimana	Ptychopteridae	6513	5+X+Y	197.9 Mb	phantom crane flies	F	(Sivell et al., 2024d)	GCA_961205885.1
Ptychoptera contaminata	Ptychopteridae	6602	4+X+Y	204.1 Mb	phantom crane flies	F		GCA_963942525.1
Rhagio lineola	Rhagionidae	7643	4+X	1.7 Gb	snipe flies	F	(Sivell et al., 2025)	GCA_964211845.1
Melanophora roralis	Rhinophoridae	8762	5+X	565.1 Mb	woodlouse flies	F	(Mitchell et al., 2024c)	GCA_963583895.1
Phyto melanocephala	Rhinophoridae	8846	5+X	739.7 Mb	woodlouse flies	F	(Mitchell et al., 2023c)	GCA_941918925.1
Sarcophaga caerulescens	Sarcophagidae	8827	5+X+Y	597.3 Mb	flesh flies	F	(Falk et al., 2023n)	GCA_927399465.1
Sarcophaga rosellei	Sarcophagidae	8839	5+X	541.4 Mb	flesh flies	F	(Falk et al., 2023o)	GCA_930367235.1
Sarcophaga subvicina	Sarcophagidae	8775	5+X	714.2 Mb	flesh flies	F	(Falk et al., 2024o)	GCA_936449025.2
Sarcophaga variegata	Sarcophagidae	8807	5+X+Y	718.5 Mb	flesh flies	F	(Falk et al., 2023e)	GCA_932273835.1
Bradysia coprophila	Sciaridae	7125	3+X	309.6 Mb	dark-winged fungus gnats	A,E	(Urban et al., 2021)	GCA_014529535.2
Pseudolycoriella hygida	Sciaridae	6907	3+X	609.3 Mb	dark-winged fungus gnats	A,E	(Trinca et al., 2023)	GCA_029228625.1
Coremacera marginata	Sciomyzidae	8966	5+X	979.7 Mb	marsh flies, snail-killing flie	F	(Sivell et al., 2021b)	GCA_914767935.1
Pherbina coryleti	Sciomyzidae	8822	5+X	863 Mb	marsh flies, snail-killing flie	F	(Sivell et al., 2023b)	GCA_943735915.1
Tetanocera ferruginea	Sciomyzidae	8946	5+X+Y	790.4 Mb	marsh flies, snail-killing flie	F	(Crowley et al., 2023d)	GCA_958299015.1
Beris chalybata	Stratiomyidae	7726	4+X+Y	541.9 Mb	soldier flies	F	(Crowley et al., 2024a)	GCA_949128065.1
Beris morrisii	Stratiomyidae	7811	4+X	613.3 Mb	soldier flies	F	(McCulloch et al., 2023)	GCA_951812415.1
Chorisops tibialis	Stratiomyidae	7818	3+X+Y	865.3 Mb	soldier flies	F	(Sivell et al., 2024a)	GCA_963669355.1
Nemotelus nigrinus	Stratiomyidae	7533	4+X	722.2 Mb	soldier flies	F	(Sivell et al., 2023c)	GCA_947369275.1
Odontomyia ornata	Stratiomyidae	7675	5+X+Y	2 Gb	soldier flies	F	(Wolton et al., 2025)	GCA_963969285.1
Blera fallax	Syrphidae	8401	5+X+Y	461.8 Mb	hover flies	F	(Taylor et al., 2025)	GCA_946965025.1
Brachypalpus laphriformis	Syrphidae	8531	4+X	1.1 Gb	hover flies	F	(Crowley et al., 2024c)	GCA_945910035.1
Cheilosia impressa	Syrphidae	8395	5+X	395 Mb	hover flies	F	(Falk et al., 2024j)	GCA_948293265.1
Cheilosia pagana	Syrphidae	8395	5+X	354.1 Mb	hover flies	F	(Falk et al., 2024k)	GCA_936431705.1
Cheilosia soror	Syrphidae	8366	4+X	470 Mb	hover flies	F	(Falk et al., 2023a)	GCA_948107745.1
Cheilosia urbana	Syrphidae	8396	4+X	546.9 Mb	hover flies	F	(Falk et al., 2024d)	GCA_946477585.1
Cheilosia vulpina	Syrphidae	8348	5+X	405.4 Mb	hover flies	F	(Falk et al., 2021b)	GCA_916610125.1
Chrysotoxum bicinctum	Syrphidae	8262	4+X	912.9 Mb	hover flies	F	(Hawkes et al., 2021a)	GCA_911387755.1
Criorhina berberina	Syrphidae	8386	3+X	410.4 Mb	hover flies	F	(Crowley et al., 2023a)	GCA_917880715.2
Dasysyrphus albostriatus	Syrphidae	8286	4+X	662.5 Mb	hover flies	F	(Crowley et al., 2024d)	GCA_946251815.1
Epistrophe grossulariae	Syrphidae	8234	4+X	556.6 Mb	hover flies	F	(Falk et al., 2023r)	GCA_929447395.1
Epistrophella euchroma	Syrphidae	8255	4+X+Y	523.3 Mb	hover flies	F	(Falk et al., 2023s)	GCA_947049315.1
Episyrphus balteatus	Syrphidae	8236	4+X	535.3 Mb	hover flies	F	(Hawkes et al., 2023b)	GCA_945859705.1
Eristalinus sepulchralis	Syrphidae	8290	5+X	376.8 Mb	hover flies	F	(Falk et al., 2023i)	GCA_944738645.1
Eristalis arbustorum	Syrphidae	8365	5+X	451 Mb	hover flies	F	(Hawkes et al., 2022a)	GCA_916610145.1
Eristalis pertinax	Syrphidae	8318	5+X+Y	482.1 Mb	hover flies	F	(Hawkes et al., 2021b)	GCA_907269125.1
Eristalis tenax	Syrphidae	8329	5+X	487 Mb	hover flies	F	(Hawkes et al., 2021c)	GCA_905231855.2
Eupeodes corollae	Syrphidae	8228	3+X	648.2 Mb	hover flies	F	(Sivell et al., 2023a)	GCA_945859685.1
Eupeodes latifasciatus	Syrphidae	8235	3+X	846.3 Mb	hover flies	F	(Falk et al., 2022)	GCA_920104205.1
Leucozona laternaria	Syrphidae	8291	4+X	613.5 Mb	hover flies	F	(Falk et al., 2023p)	GCA_932273885.2
Melanostoma mellinum	Syrphidae	8383	3+X+Y	731 Mb	hover flies	F	(Hawkes et al., 2022b)	GCA_914767635.1
Meliscaeva auricollis	Syrphidae	8198	4+X	385.1 Mb	hover flies	F	(Falk et al., 2024q)	GCA_948107695.1
Myathropa florea	Syrphidae	8352	4+X+Y	485.1 Mb	hover flies	F	(Hawkes et al., 2023c)	GCA_930367185.1
Neoascia interrupta	Syrphidae	8430	3+X	601.9 Mb	hover flies	F	(Falk et al., 2023t)	GCA_947623515.1
Platycheirus albimanus	Syrphidae	8246	3+X	677.8 Mb	hover flies	F	(Crowley et al., 2023i)	GCA_916050605.2
Rhingia campestris	Syrphidae	8430	4+X	719.9 Mb	hover flies	F	(Crowley et al., 2023e)	GCA_932526625.1
Scaeva pyrastri	Syrphidae	8092	3+X	320 Mb	hover flies	F	(Hawkes et al., 2023a)	GCA_905146935.1
Sphaerophoria taeniata	Syrphidae	8252	3+X+Y	609.3 Mb	hover flies	F	(Falk et al., 2023m)	GCA_943590905.1
Syritta pipiens	Syrphidae	8382	4+X	318.5 Mb	hover flies	F	(Crowley et al., 2023c)	GCA_905187475.1
Volucella inanis	Syrphidae	8372	5+X	961.4 Mb	hover flies	F	(Crowley et al., 2023f)	GCA_907269105.1
Volucella inflata	Syrphidae	8345	5+X	753.5 Mb	hover flies	F	(Crowley et al., 2023h)	GCA_928272305.1
Xanthogramma pedissequum	Syrphidae	8175	4+X+Y	977.2 Mb	hover flies	F	(Sivell et al., 2022)	GCA_910595825.1
Xylota sylvarum	Syrphidae	8422	4+X	534.8 Mb	hover flies	F	(Crowley et al., 2023g)	GCA_905220385.1
Cistogaster globosa	Tachinidae	8715	5+X+Y	837.8 Mb	parasitic flies	F	(Falk et al., 2023c)	GCA_937654795.1
Cochliomyia hominivorax	Tachinidae	8797	5+X	534 Mb	parasitic flies	F	(Tandonnet et al., 2023)	
Epicampocera succincta	Tachinidae	8499	5+X+Y	398.1 Mb	parasitic flies	F	(Falk et al., 2023f)	GCA_932526305.1
Gymnosoma rotundatum	Tachinidae	8742	5+X	779.1 Mb	parasitic flies	F	(Smith et al., 2022)	GCA_916610165.2

Lypha dubia	Tachinidae	8693	5+X	645 Mb	parasitic flies	F	(Falk et al., 2024a)	GCA_947311025.1
Nowickia ferox	Tachinidae	8717	5+X	670.7 Mb	parasitic flies	F	(Falk et al., 2023g)	GCA_936439885.1
Tachina fera	Tachinidae	8734	5+X	751.7 Mb	parasitic flies	F	(University of Oxford and \	GCA_905220375.1
Tachina lurida	Tachinidae	8746	5+X	899.2 Mb	parasitic flies	F	(Falk et al., 2023k)	GCA_944452675.1
Thecocarcelia acutangulata	Tachinidae	8571	5+X	692.9 Mb	parasitic flies	F	(Falk et al., 2023q)	GCA_914767995.1
Thelaira solivaga	Tachinidae	8562	5+X+Y	429.3 Mb	parasitic flies	F	(Falk et al., 2023l)	GCA_947397855.1
Anastrepha ludens	Tephritidae	8900	5+X+Y	820.8 Mb	fruit flies	F	(Congrains et al., 2024)	GCA_028408465.1
Thereva nobilitata	Therevidae	7753	5+X	29.2 Mb	stiletto flies	F	(McCulloch et al., 2025b)	GCA_963855945.1
Nephrotoma appendiculata	Tipulidae	7147	3+X	1.1 Gb	large crane flies	F	(Crowley et al., 2024e)	GCA_947310385.1
Nephrotoma flavescens	Tipulidae	7145	3+X	1.1 Gb	large crane flies	F	(Sivell et al., 2023e)	GCA_932526605.1

**Assigned according to Ellison C, Bachtrog D (2019) Recurrent gene co-amplification on Drosophila X and Y chromosomes. PLoS Genet 15(7): e1008251. https://doi.org/10.1371/journal.pgen.1008251*

***Assigned according to Vicoso B, Bachtrog D (2015) Numerous Transitions of Sex Chromosomes in Diptera. PLOS Biology 13(4): e1002078. https://doi.org/10.1371/journal.pbio.1002078*

References:

Alencar, R.M., Sepulveda, C.C.P., Martinez-Villegas, L., Bahia, A.C., Santana, R.A., De Souza, I.B., *et al.* (2023) Unravelling the genome of the brackish water malaria vector Anopheles aquasalis. *Scientific Reports*, **13**, 20472.

Ashworth, M., Falk, S., Natural History Museum Genome Acquisition Lab, University of Oxford and Wytham Woods Genome Acquisition Lab, Darwin Tree of Life Barcoding collective, Wellcome Sanger Institute Tree of Life programme, *et al.* (2023) The genome sequence of the thick-headed fly, Myopa tessellatipennis (Motschulsky, 1859). *Wellcome Open Research*, **8**, 115.

Ashworth, M., McCulloch, J., Crowley, L.M., Drake, C.M., Natural History Museum Genome Acquisition Lab, University of Oxford and Wytham Woods Genome Acquisition Lab, *et al.* (2025) The genome sequence of Dolichopus griseipennis Stannius, 1831. *Wellcome Open Research*, **10**, 29.

Ayala, D., Akone-Ella, O., Kengne, P., Johnson, H., Heaton, H., Collins, J., *et al.* (2023) The genome sequence of the malaria mosquito, Anopheles funestus, Giles, 1900. *Wellcome Open Research*, **7**, 287.

Barclay, M.V.L., Broad, G.R., Sivell, O., Natural History Museum Genome Acquisition Lab, Darwin Tree of Life Barcoding collective, Wellcome Sanger Institute Tree of Life Management, Samples and Laboratory team, *et al.* (2025) The genome sequence of a pallopterid fly, Toxonevra muliebris (Harris, 1780). *Wellcome Open Research*, **10**, 55.

Bouafou, L.B.A., Ayala, D., Makanga, B.K., Rahola, N., Johnson, H.F., Heaton, H., *et al.* (2024) Chromosomal reference genome sequences for the malaria mosquito, Anopheles coustani, Laveran, 1900. *Wellcome Open Research*, **9**, 551.

Bracewell, R., Chatla, K., Nalley, M.J. & Bachtrog, D. (2019) Dynamic turnover of centromeres drives karyotype evolution in Drosophila. *eLife*, **8**, e49002.

Broad, G.R., Natural History Museum Genome Acquisition Lab, Darwin Tree of Life Barcoding collective, Wellcome Sanger Institute Tree of Life programme, Wellcome Sanger Institute Scientific Operations: DNA Pipelines collective, Tree of Life Core Informatics collective, *et al.* (2022) The genome sequence of the dotted bee-fly, Bombylius discolor (Mikan, 1796). *Wellcome Open Research*, **7**, 306.

Butlin, R., Mérot, C., Wellcome Sanger Institute Tree of Life Management, Samples and Laboratory team, Wellcome Sanger Institute Scientific Operations: Sequencing Operations, Wellcome Sanger Institute Tree of Life Core Informatics team, Tree of Life Core Informatics collective, *et al.* (2024) The genome sequence of a kelp fly, Coelopa pilipes Haliday, 1838. *Wellcome Open Research*, **9**, 197.

Chakraborty, M., Chang, C.-H., Khost, D.E., Vedanayagam, J., Adrion, J.R., Liao, Y., *et al.* (2021) Evolution of genome structure in the *Drosophila simulans* species complex. *Genome Research*, **31**, 380–396.

Congrains, C., Sim, S.B., Paulo, D.F., Corpuz, R.L., Kauwe, A.N., Simmonds, T.J., *et al.* (2024) Chromosome-scale genome of the polyphagous pest *Anastrepha ludens* (Diptera: Tephritidae) provides insights on sex chromosome evolution in *Anastrepha*. *G3: Genes, Genomes, Genetics*, jkae239.

Crowley, L., University of Oxford and Wytham Woods Genome Acquisition Lab, Darwin Tree of Life Barcoding collective, Wellcome Sanger Institute Tree of Life programme, Wellcome Sanger Institute Scientific Operations: DNA Pipelines collective, Tree of Life Core Informatics collective, *et al.* (2023a) The genome sequence of the Dimorphic Bear Hoverfly, Criorhina berberina (Fabricius, 1805). *Wellcome Open Research*, **8**, 23.

Crowley, L.M., Akinmusola, R.Y., University of Oxford and Wytham Woods Genome Acquisition Lab, Darwin Tree of Life Barcoding collective, Wellcome Sanger Institute Tree of Life Management, Samples and Laboratory team, Wellcome Sanger Institute Scientific Operations: Sequencing Operations, *et al.* (2024a) The genome sequence of the yellow-legged black legionnaire, Beris chalybata (Forster, 1771). *Wellcome Open Research*, **9**, 151.

Crowley, L.M., Akinmusola, R.Y., University of Oxford and Wytham Woods Genome Acquisition Lab, Darwin Tree of Life Barcoding collective, Wellcome Sanger Institute Tree of Life programme, Wellcome Sanger Institute Scientific Operations: DNA Pipelines collective, *et al.* (2023b) The genome sequence of a druid fly, Clusia tigrina (Fallén, 1820). *Wellcome Open Research*, **8**, 430.

Crowley, L.M., Ashworth, M., Wawman, D.C., University of Oxford and Wytham Woods Genome Acquisition Lab, Natural History Museum Genome Acquisition Lab, Darwin Tree of Life Barcoding collective, *et al.* (2023c) The genome sequence of the Thick-legged Hoverfly, Syritta pipiens (Linnaeus, 1758). *Wellcome Open Research*, **8**, 349.

Crowley, L.M., Falk, S., Williams, C.D., University of Oxford and Wytham Woods Genome Acquisition Lab, Darwin Tree of Life Barcoding collective, Wellcome Sanger Institute Tree of Life programme, *et al.* (2023d) The genome sequence of the common buff snailkiller, Tetanocera ferruginea (Fallén, 1820). *Wellcome Open Research*, **8**, 535.

Crowley, L.M., Mitchell, R., University of Oxford and Wytham Woods Genome Acquisition Lab, Darwin Tree of Life Barcoding collective, Wellcome Sanger Institute Tree of Life programme, Wellcome Sanger Institute Scientific Operations: DNA Pipelines collective, *et al.* (2023e) The genome sequence of the Common Snout

Hoverfly, Rhingia campestris (Meigen, 1822). *Wellcome Open Research*, **8**, 100.
Crowley, L.M., Mitchell, R., University of Oxford and Wytham Woods Genome Acquisition Lab, Natural History Museum Genome Acquisition Lab, Darwin Tree of Life Barcoding collective, Wellcome Sanger Institute Tree of Life programme, *et al.* (2023f) The genome sequence of the Lesser Hornet Hoverfly, Volucella inanis (Linnaeus, 1758). *Wellcome Open Research*, **8**, 69.
Crowley, L.M., Nash, W., University of Oxford and Wytham Woods Genome Acquisition Lab, Darwin Tree of Life Barcoding collective, Wellcome Sanger Institute Tree of Life programme, Wellcome Sanger Institute Scientific Operations: DNA Pipelines collective, *et al.* (2023g) The genome sequence of the Golden-tailed Leafwalker, Xylota sylvarum (Linnaeus, 1758). *Wellcome Open Research*, **8**, 134.
Crowley, L.M., Phillips, N., Hardy, E., McAlister, E., University of Oxford and Wytham Woods Genome Acquisition Lab, Natural History Museum Genome Acquisition Lab, *et al.* (2024b) The genome sequence of the Top-horned Hunchback fly, Acrocera orbiculus (Fabricius, 1787). *Wellcome Open Research*, **9**, 25.
Crowley, L.M., Poole, O., University of Oxford and Wytham Woods Genome Acquisition Lab, Darwin Tree of Life Barcoding collective, Wellcome Sanger Institute Tree of Life Management, Samples and Laboratory team, Wellcome Sanger Institute Scientific Operations: Sequencing Operations, *et al.* (2024c) The genome sequence of the forest hoverfly, Brachypalpus laphriformis (Fallén, 1816). *Wellcome Open Research*, **9**, 39.
Crowley, L.M., Sivell, O., Sivell, D., Mitchell, R., Barnes, J.C., Wotton, K.R., *et al.* (2023h) The genome sequence of the Cossus hoverfly, Volucella inflata (Fabricius 1794). *Wellcome Open Research*, **8**, 465.
Crowley, L.M., Wawman, D.C., University of Oxford and Wytham Woods Genome Acquisition Lab, Darwin Tree of Life Barcoding collective, Wellcome Sanger Institute Tree of Life Management, Samples and Laboratory team, Wellcome Sanger Institute Scientific Operations: Sequencing Operations, *et al.* (2024d) The genome sequence of the Stripe-backed Dasysyrphus, Dasysyrphus albostriatus (Fallén, 1817). *Wellcome Open Research*, **9**, 34.
Crowley, L.M., Wawman, D.C., University of Oxford and Wytham Woods Genome Acquisition Lab, Darwin Tree of Life Barcoding collective, Wellcome Sanger Institute Tree of Life Management, Samples and Laboratory team, Wellcome Sanger Institute Scientific Operations: Sequencing Operations, *et al.* (2024e) The genome sequence of the spotted cranefly, Nephrotoma appendiculata (Pierre, 1919). *Wellcome Open Research*, **9**, 38.
Crowley, L.M., Woodcock, K.J., University of Oxford and Wytham Woods Genome Acquisition Lab, Darwin Tree of Life Barcoding collective, Wellcome Sanger Institute Tree of Life Management, Samples and Laboratory team, Wellcome Sanger Institute Scientific Operations: Sequencing Operations, *et al.* (2023i) The genome sequence of the white-footed hoverfly, Platycheirus albimanus (Fabricius, 1781). *Wellcome Open Research*, **8**, 572.
Durkin, S.M., Chakraborty, M., Abrieux, A., Lewald, K.M., Gadau, A., Svetec, N., *et al.* (2021) Behavioral and Genomic Sensory Adaptations Underlying the Pest Activity of *Drosophila suzukii*. *Molecular Biology and Evolution*, **38**, 2532–2546.
Falk, S., Akinmusola, R.Y., University of Oxford and Wytham Woods Genome Acquisition Lab, Darwin Tree of Life Barcoding collective, Wellcome Sanger Institute Tree of Life Management, Samples and Laboratory team, Wellcome Sanger Institute Scientific Operations: Sequencing Operations, *et al.* (2024a) The genome sequence of a tachinid fly, Lypha dubia Fallén, 1810. *Wellcome Open Research*, **9**, 345.
Falk, S., Brighton, P., University of Oxford and Wytham Woods Genome Acquisition Lab, Darwin Tree of Life Barcoding collective, Wellcome Sanger Institute Tree of Life Management, Samples and Laboratory team, Wellcome Sanger Institute Scientific Operations: Sequencing Operations, *et al.* (2024b) The genome sequence of an anthomyiid fly, Pegoplata infirma (Meigen, 1826) (Diptera, Anthomyiidae). *Wellcome Open Research*, **9**, 330.
Falk, S., Crowley, L.M., Akinmusola, R.Y., University of Oxford and Wytham Woods Genome Acquisition Lab, Darwin Tree of Life Barcoding collective, Wellcome Sanger Institute Tree of Life Management, Samples and Laboratory team, *et al.* (2025a) The genome sequence of the muscid fly Eudasyphora cyanicolor (Zetterstedt, 1845). *Wellcome Open Research*, **10**, 56.
Falk, S., Crowley, L.M., Clements, D.K., University of Oxford and Wytham Woods Genome Acquisition Lab, Darwin Tree of Life Barcoding collective, Wellcome Sanger Institute Tree of Life Management, Samples and Laboratory team, *et al.* (2024c) The genome sequence of the Four-banded Bee-grabber, Conops quadrifasciatus De Geer, 1776. *Wellcome Open Research*, **9**, 136.
Falk, S., Crowley, L.M., Poole, O., University of Oxford and Wytham Woods Genome Acquisition Lab, Darwin Tree of Life Barcoding collective, Wellcome Sanger Institute Tree of Life programme, *et al.* (2023a) The genome sequence of the Truffle Blacklet, Cheilosia soror (Zetterstedt, 1843). *Wellcome Open Research*, **8**, 443.
Falk, S., Darwin Tree of Life Barcoding collective, Wellcome Sanger Institute Tree of Life programme, Wellcome Sanger Institute Scientific Operations: DNA Pipelines collective, Tree of Life Core Informatics collective, Sivell, O., *et al.* (2023b) The genome sequence of a bird blowfly, Protocalliphora azurea

(Fallén,1817). *Wellcome Open Research*, **8**, 67.
Falk, S., Gorše, I., University of Oxford and Wytham Woods Genome Acquisition Lab, Darwin Tree of Life Barcoding collective, Wellcome Sanger Institute Tree of Life programme, Wellcome Sanger Institute Scientific Operations: DNA Pipelines collective, *et al.* (2024d) The genome sequence of the hawkweed Cheilosia, Cheilosia urbana (Meigen, 1822). *Wellcome Open Research*, **8**, 311.
Falk, S., Grzywacz, A., University of Oxford and Wytham Woods Genome Acquisition Lab, Darwin Tree of Life Barcoding collective, Wellcome Sanger Institute Tree of Life Management, Samples and Laboratory team, Wellcome Sanger Institute Scientific Operations: Sequencing Operations, *et al.* (2024e) The genome sequence of a muscid fly, Hydrotaea cyrtoneurina (Zetterstedt, 1845). *Wellcome Open Research*, **9**, 60.
Falk, S., Grzywacz, A., University of Oxford and Wytham Woods Genome Acquisition Lab, Darwin Tree of Life Barcoding collective, Wellcome Sanger Institute Tree of Life Management, Samples and Laboratory team, Wellcome Sanger Institute Scientific Operations: Sequencing Operations, *et al.* (2024f) The genome sequence of a muscid fly, Hydrotaea diabolus (Harris, [1780]). *Wellcome Open Research*, **9**, 176.
Falk, S., Grzywacz, A., University of Oxford and Wytham Woods Genome Acquisition Lab, Darwin Tree of Life Barcoding collective, Wellcome Sanger Institute Tree of Life Management, Samples and Laboratory team, Wellcome Sanger Institute Scientific Operations: Sequencing Operations, *et al.* (2024g) The genome sequence of a muscid fly, Muscina levida (Harris, 1780). *Wellcome Open Research*, **9**, 537.
Falk, S., Lennon, R., University of Oxford Genome and Wytham Woods Genome Acquisition Lab, Darwin Tree of Life Barcoding collective, Wellcome Sanger Institute Tree of Life programme, Wellcome Sanger Institute Scientific Operations: DNA Pipelines collective, *et al.* (2023c) The genome sequence of a tachinid fly, Cistogaster globosa (Fabricius, 1775). *Wellcome Open Research*, **8**, 462.
Falk, S., Mallick, T., University of Oxford and Wytham Woods Genome Acquisition Lab, Darwin Tree of Life Barcoding collective, Wellcome Sanger Institute Tree of Life Management, Samples and Laboratory team, Wellcome Sanger Institute Scientific Operations: Sequencing Operations, *et al.* (2024h) The genome sequence of a dance fly, Empis livida Linnaeus, 1758. *Wellcome Open Research*, **9**, 497.
Falk, S., McCulloch, J., University of Oxford and Wytham Woods Genome Acquisition Lab, Darwin Tree of Life Barcoding collective, Wellcome Sanger Institute Tree of Life Management, Samples and Laboratory team, Wellcome Sanger Institute Scientific Operations: Sequencing Operations, *et al.* (2025b) The genome sequence of the fever fly, Dilophus febrilis (Linnaeus, 1758). *Wellcome Open Research*, **10**, 21.
Falk, S., Mitchell, R., Sivell, O., University of Oxford and Wytham Woods Genome Acquisition Lab, Natural History Museum Genome Acquisition Lab, Darwin Tree of Life Barcoding collective, *et al.* (2023d) The genome sequence of the black-bellied cluster fly, Pollenia amentaria (Scopoli, 1763). *Wellcome Open Research*, **8**, 466.
Falk, S., Mulley, J.F., University of Oxford and Wytham Woods Genome Acquisition Lab, Darwin Tree of Life Barcoding collective, Wellcome Sanger Institute Tree of Life programme, Wellcome Sanger Institute Scientific Operations: DNA Pipelines collective, *et al.* (2023e) The genome sequence of the variegated flesh fly, Sarcophaga variegata (Scopoli, 1763). *Wellcome Open Research*, **8**, 234.
Falk, S., Poole, O., University of Oxford and Wytham Woods Genome Acquisition Lab, Darwin Tree of Life Barcoding collective, Wellcome Sanger Institute Tree of Life Management, Samples and Laboratory team, Wellcome Sanger Institute Scientific Operations: Sequencing Operations, *et al.* (2024i) The genome sequence of a conopid fly, Myopa testacea (Linnaeus, 1767). *Wellcome Open Research*, **9**, 99.
Falk, S., Poole, O., University of Oxford and Wytham Woods Genome Acquisition Lab, Darwin Tree of Life Barcoding collective, Wellcome Sanger Institute Tree of Life Management, Samples and Laboratory team, Wellcome Sanger Institute Scientific Operations: Sequencing Operations, *et al.* (2024j) The genome sequence of a hoverfly, Cheilosia impressa (Loew, 1840). *Wellcome Open Research*, **9**, 74.
Falk, S., Poole, O., University of Oxford and Wytham Woods Genome Acquisition Lab, Darwin Tree of Life Barcoding collective, Wellcome Sanger Institute Tree of Life Management, Samples and Laboratory team, Wellcome Sanger Institute Scientific Operations: Sequencing Operations, *et al.* (2024k) The genome sequence of the parsley Cheilosia, Cheilosia pagana (Meigen, 1822). *Wellcome Open Research*, **9**, 54.
Falk, S., Raper, C., University of Oxford and Wytham Woods Genome Acquisition Lab, Darwin Tree of Life Barcoding collective, Wellcome Sanger Institute Tree of Life programme, Wellcome Sanger Institute Scientific Operations: DNA Pipelines collective, *et al.* (2023f) The genome sequence of the tachinid fly, Epicampocera succincta (Meigen, 1824). *Wellcome Open Research*, **8**, 276.
Falk, S., Raper, C., University of Oxford and Wytham Woods Genome Acquisition Lab, Darwin Tree of Life Barcoding collective, Wellcome Sanger Institute Tree of Life programme, Wellcome Sanger Institute Scientific Operations: DNA Pipelines collective, *et al.* (2023g) The genome sequence of a tachinid fly, Nowickia ferox (Panzer, 1809). *Wellcome Open Research*, **8**, 275.
Falk, S., Sivell, D., Badham, X.R., University of Oxford and Wytham Woods Genome Acquisition Lab, Natural History Museum Genome Acquisition Lab, Darwin Tree of Life Barcoding collective, *et al.* (2023h) The genome sequence of a heleomyzid fly, Suillia variegata (Loew, 1862). *Wellcome Open Research*, **8**,

382.
Falk, S., Sivell, D., Webb, J., Grzywacz, A., University of Oxford and Wytham Woods Genome Acquisition Lab, Natural History Museum Genome Acquisition Lab, *et al.* (2024l) The genome sequence of a muscid fly, Polietes domitor (Harris, 1780). *Wellcome Open Research*, **9**, 58.
Falk, S., Sivell, O., Sivell, D., Mitchell, R., Woodcock, K.J., University of Oxford and Wytham Woods Genome Acquisition Lab, *et al.* (2023i) The genome sequence of the small spotty-eyed dronefly, Eristalinus sepulchralis (Linnaeus, 1758). *Wellcome Open Research*, **8**, 269.
Falk, S., Sivell, O., University of Oxford and Wytham Woods Genome Acquisition Lab, Darwin Tree of Life Barcoding collective, Wellcome Sanger Institute Tree of Life programme, Wellcome Sanger Institute Scientific Operations: DNA Pipelines collective, *et al.* (2023j) The genome sequence of the narrow-cheeked clusterfly, Pollenia angustigena (Wainwright, 1940). *Wellcome Open Research*, **8**, 270.
Falk, S., Smith, M.N., University of Oxford and Wytham Woods Genome Acquisition Lab, Darwin Tree of Life Barcoding collective, Wellcome Sanger Institute Tree of Life programme, Wellcome Sanger Institute Scientific Operations: DNA Pipelines collective, *et al.* (2023k) The genome sequence of a tachinid fly, Tachina lurida (Fabricius, 1781). *Wellcome Open Research*, **8**, 288.
Falk, S., Smith, M.N., University of Oxford and Wytham Woods Genome Acquisition Lab, Darwin Tree of Life Barcoding collective, Wellcome Sanger Institute Tree of Life programme, Wellcome Sanger Institute Scientific Operations: DNA Pipelines collective, *et al.* (2023l) The genome sequence of a tachinid fly, Thelaira solivaga (Harris, 1780). *Wellcome Open Research*, **8**, 564.
Falk, S., Sudworth, J., University of Oxford and Wytham Woods Genome Acquisition Lab, Darwin Tree of Life Barcoding collective, Wellcome Sanger Institute Tree of Life programme, Wellcome Sanger Institute Scientific Operations: DNA Pipelines collective, *et al.* (2023m) The genome sequence of a hoverfly, Sphaerophoria taeniata (Meigen, 1822). *Wellcome Open Research*, **8**, 474.
Falk, S., University of Oxford and Wytham Woods Genome Acquisition Lab, Darwin Tree of Life Barcoding collective, Wellcome Sanger Institute Tree of Life Management, Samples and Laboratory team, Wellcome Sanger Institute Scientific Operations: Sequencing Operations, Wellcome Sanger Institute Tree of Life Core Informatics team, *et al.* (2024m) The genome sequence of a dance fly, Rhamphomyia laevipes (Fallén, 1816). *Wellcome Open Research*, **9**, 462.
Falk, S., University of Oxford and Wytham Woods Genome Acquisition Lab, Darwin Tree of Life Barcoding collective, Wellcome Sanger Institute Tree of Life Management, Samples and Laboratory team, Wellcome Sanger Institute Scientific Operations: Sequencing Operations, Wellcome Sanger Institute Tree of Life Core Informatics team, *et al.* (2024n) The genome sequence of a lauxaniid fly, Calliopum simillimum (Collin, 1933). *Wellcome Open Research*, **9**, 675.
Falk, S., University of Oxford and Wytham Woods Genome Acquisition Lab, Darwin Tree of Life Barcoding collective, Wellcome Sanger Institute Tree of Life programme, Wellcome Sanger Institute Scientific Operations: DNA Pipelines collective, Tree of Life Core Informatics collective, *et al.* (2021a) The genome sequence of the bisetose emerald-bottle, Bellardia pandia (Walker, 1849). *Wellcome Open Research*, **6**, 344.
Falk, S., University of Oxford and Wytham Woods Genome Acquisition Lab, Darwin Tree of Life Barcoding collective, Wellcome Sanger Institute Tree of Life programme, Wellcome Sanger Institute Scientific Operations: DNA Pipelines collective, Tree of Life Core Informatics collective, *et al.* (2022) The genome sequence of the meadow field syrph, Eupeodes latifasciatus (Macquart, 1829). *Wellcome Open Research*, **7**, 253.
Falk, S., University of Oxford and Wytham Woods Genome Acquisition Lab, Darwin Tree of Life Barcoding collective, Wellcome Sanger Institute Tree of Life programme, Wellcome Sanger Institute Scientific Operations: DNA Pipelines collective, Tree of Life Core Informatics collective, *et al.* (2023n) The genome sequence of the bluish flesh fly, Sarcophaga (Robineauella) caerulescens (Zetterstedt, 1838). *Wellcome Open Research*, **8**, 17.
Falk, S., University of Oxford and Wytham Woods Genome Acquisition Lab, Darwin Tree of Life Barcoding collective, Wellcome Sanger Institute Tree of Life programme, Wellcome Sanger Institute Scientific Operations: DNA Pipelines collective, Tree of Life Core Informatics collective, *et al.* (2023o) The genome of Roselle's flesh fly Sarcophaga (Helicophagella) rosellei (Böttcher, 1912). *Wellcome Open Research*, **8**, 43.
Falk, S., University of Oxford and Wytham Woods Genome Acquisition Lab, Darwin Tree of Life Barcoding collective, Wellcome Sanger Institute Tree of Life programme, Wellcome Sanger Institute Scientific Operations: DNA Pipelines collective, Tree of Life Core Informatics collective, *et al.* (2023p) The genome sequence of the dark-saddled leucozona, Leucozona laternaria (Muller, 1776). *Wellcome Open Research*, **8**, 10.
Falk, S., University of Oxford and Wytham Woods Genome Acquisition Lab, Darwin Tree of Life Barcoding collective, Wellcome Sanger Institute Tree of Life programme, Wellcome Sanger Institute Scientific Operations: DNA Pipelines collective, Tree of Life Core Informatics collective, *et al.* (2023q) The genome

sequence of a tachinid fly, Thecocarcelia acutangulata (Macquart, 1850). *Wellcome Open Research*, **8**, 88.
Falk, S., University of Oxford and Wytham Woods Genome Acquisition Lab, Darwin Tree of Life Barcoding collective, Wellcome Sanger Institute Tree of Life programme, Wellcome Sanger Institute Scientific Operations: DNA Pipelines collective, Tree of Life Core Informatics collective, *et al.* (2024o) The genome sequence of the lesser worm flesh fly, Sarcophaga (Sarcophaga) subvicina Rohdendorf, 1937. *Wellcome Open Research*, **8**, 65.
Falk, S., University of Oxford and Wytham Woods Genome Acquisition Lab, Natural History Museum Genome Acquisition Lab, Darwin Tree of Life Barcoding collective, Wellcome Sanger Institute Tree of Life programme, Wellcome Sanger Institute Scientific Operations: DNA Pipelines collective, *et al.* (2021b) The genome sequence of the large burdock Cheilosia, Cheilosia vulpina (Meigen, 1822). *Wellcome Open Research*, **6**, 351.
Falk, S., Wawman, D.C., University of Oxford and Wytham Woods Genome Acquisition Lab, Darwin Tree of Life Barcoding collective, Wellcome Sanger Institute Tree of Life Management, Samples and Laboratory team, Wellcome Sanger Institute Scientific Operations: Sequencing Operations, *et al.* (2024p) The genome sequence of a pipunculid fly, Nephrocerus scutellatus (Macquart, 1834). *Wellcome Open Research*, **9**, 41.
Falk, S., Wolton, R., Mitchell, R., Woodcock, K.J., University of Oxford and Wytham Woods Genome Acquisition Lab, Natural History Museum Genome Acquisition Lab, *et al.* (2023r) The genome sequence of the broad-banded Epistrophe, Epistrophe grossulariae (Meigen, 1822). *Wellcome Open Research*, **8**, 438.
Falk, S., Woodcock, K.J., University of Oxford and Wytham Woods Genome Acquisition Lab, Darwin Tree of Life Barcoding collective, Wellcome Sanger Institute Tree of Life Management, Samples and Laboratory team, Wellcome Sanger Institute Scientific Operations: Sequencing Operations, *et al.* (2024q) The genome sequence of the spotted Meliscaeva, Meliscaeva auricollis (Meigen, 1822). *Wellcome Open Research*, **9**, 82.
Falk, S., Woodcock, K.J., University of Oxford and Wytham Woods Genome Acquisition Lab, Darwin Tree of Life Barcoding collective, Wellcome Sanger Institute Tree of Life programme, Wellcome Sanger Institute Scientific Operations: DNA Pipelines collective, *et al.* (2023s) The genome sequence of the hoverfly, Epistrophella euchroma (Kowarz, 1885). *Wellcome Open Research*, **8**, 279.
Falk, S., Woodcock, K.J., University of Oxford and Wytham Woods Genome Acquisition Lab, Darwin Tree of Life Barcoding collective, Wellcome Sanger Institute Tree of Life programme, Wellcome Sanger Institute Scientific Operations: DNA Pipelines collective, *et al.* (2023t) The genome sequence of the bulrush Neoascia, Neoascia interrupta (Meigen, 1822). *Wellcome Open Research*, **8**, 549.
Gendrin, M., Heu, K., Moreno, M., Gamboa, D., Vinetz, J.M., Tong, C., *et al.* (2025) Two chromosomal reference genome sequences for the malaria mosquito, Anopheles (Nyssorhynchus) darlingi, Root, 1926 from French Guiana and Peru. *Wellcome Open Research*, **10**, 187.
Habtewold, T., Wagah, M., Tambwe, M.M., Moore, S., Windbichler, N., Christophides, G., *et al.* (2023) A chromosomal reference genome sequence for the malaria mosquito, Anopheles gambiae, Giles, 1902, Ifakara strain. *Wellcome Open Research*, **8**, 74.
Hawkes, W., Sivell, O., Sivell, D., University of Oxford and Wytham Woods Genome Acquisition Lab, Darwin Tree of Life Barcoding collective, Wellcome Sanger Institute Tree of Life programme, *et al.* (2023a) The genome sequence of the pied hoverfly, Scaeva pyrastri (Linnaeus, 1758). *Wellcome Open Research*, **8**, 83.
Hawkes, W., Wotton, K., Smith, M., University of Oxford and Wytham Woods Genome Acquisition Lab, Natural History Museum Genome Acquisition Lab, Darwin Tree of Life Barcoding collective, *et al.* (2021a) The genome sequence of the two-banded wasp hoverfly, Chrysotoxum bicinctum (Linnaeus, 1758). *Wellcome Open Research*, **6**, 321.
Hawkes, W., Wotton, K., University of Oxford and Wytham Woods Genome Acquisition Lab, Darwin Tree of Life Barcoding collective, Wellcome Sanger Institute Tree of Life programme, Wellcome Sanger Institute Scientific Operations: DNA Pipelines collective, *et al.* (2021b) The genome sequence of the tapered dronefly, Eristalis pertinax (Scopoli, 1763). *Wellcome Open Research*, **6**, 292.
Hawkes, W., Wotton, K., University of Oxford and Wytham Woods Genome Acquisition Lab, Darwin Tree of Life Barcoding collective, Wellcome Sanger Institute Tree of Life programme, Wellcome Sanger Institute Scientific Operations: DNA Pipelines collective, *et al.* (2021c) The genome sequence of the drone fly, Eristalis tenax (Linnaeus, 1758). *Wellcome Open Research*, **6**, 307.
Hawkes, W., Wotton, K., University of Oxford and Wytham Woods Genome Acquisition Lab, Darwin Tree of Life Barcoding collective, Wellcome Sanger Institute Tree of Life programme, Wellcome Sanger Institute Scientific Operations: DNA Pipelines collective, *et al.* (2022a) The genome sequence of the plain-faced dronefly, Eristalis arbustorum (Linnaeus, 1758). *Wellcome Open Research*, **7**, 61.
Hawkes, W., Wotton, K., University of Oxford and Wytham Woods Genome Acquisition Lab, Darwin Tree of Life Barcoding collective, Wellcome Sanger Institute Tree of Life programme, Wellcome Sanger Institute Scientific Operations: DNA Pipelines collective, *et al.* (2022b) The genome sequence of the dumpy grass

hoverfly, Melanostoma mellinum (Linnaeus, 1758). *Wellcome Open Research*, **7**, 59.
Hawkes, W.L., Sivell, O., University of Oxford and Wytham Woods Genome Acquisition Lab, Natural History Museum Genome Acquisition Lab, Darwin Tree of Life Barcoding collective, Wellcome Sanger Institute Tree of Life programme, *et al.* (2023b) The genome sequence of the Marmalade Hoverfly, Episyrphus balteatus (De Geer, 1776). *Wellcome Open Research*, **8**, 106.
Hawkes, W.L.S., Wotton, K.R., University of Oxford and Wytham Woods Genome Acquisition Lab, Darwin Tree of Life Barcoding collective, Wellcome Sanger Institute Tree of Life programme, Wellcome Sanger Institute Scientific Operations: DNA Pipelines collective, *et al.* (2023c) The genome sequence of the Batman Hoverfly, Myathropa florea (Linnaeus, 1758). *Wellcome Open Research*, **8**, 470.
Hemmer, L.W., Dias, G.B., Smith, B., Van Vaerenberghe, K., Howard, A., Bergman, C.M., *et al.* (2020) Hybrid dysgenesis in Drosophila virilis results in clusters of mitotic recombination and loss-of-heterozygosity but leaves meiotic recombination unaltered. *Mobile DNA*, **11**, 10.
Hill, T., Koseva, B.S. & Unckless, R.L. (2019) The Genome of Drosophila innubila Reveals Lineage-Specific Patterns of Selection in Immune Genes. *Molecular Biology and Evolution*, **36**, 1405–1417.
Hoskins, R. A., Carlson, J. W., Wan, K. H., Park, S., Mendez, I., Galle, S. E., Booth, B. W., Pfeiffer, B. D., George, R. A., Svirskas, R., Krzywinski, M., Schein, J., Accardo, M. C., Damia, E., Messina, G., Méndez-Lago, M., de Pablos, B., Demakova, O. V., Andreyeva, E. N., Boldyreva, L. V., … Celniker, S. E. (2015). The Release 6 reference sequence of the Drosophila melanogaster genome. *Genome research*, ***25***(3), 445–458. https://doi.org/10.1101/gr.185579.114
Konganti, K., Guerrero, F.D., Schilkey, F., Ngam, P., Jacobi, J.L., Umale, P.E., *et al.* (2018) A Whole Genome Assembly of the Horn Fly, *Haematobia irritans* , and Prediction of Genes with Roles in Metabolism and Sex Determination. *G3 Genes|Genomes|Genetics*, **8**, 1675–1686.
Lawniczak, M.K.N., Crowley, L.M., McAlister, E., University of Oxford and Wytham Woods Genome Acquisition Lab, Darwin Tree of Life Barcoding collective, Wellcome Sanger Institute Tree of Life programme, *et al.* (2023) The genome sequence of the dark-edged bee fly, Bombylius major (Linnaeus, 1758). *Wellcome Open Research*, **8**, 379.
Leung, W., Torosin, N., Cao, W., Reed, L.K., Arrigo, C., Elgin, S.C.R., *et al.* (2023) Long-read genome assemblies for the study of chromosome expansion: *Drosophila kikkawai* , *Drosophila takahashii* , *Drosophila bipectinata* , and *Drosophila ananassae*. *G3: Genes, Genomes, Genetics*, **13**, jkad191.
Lukyanchikova, V., Nuriddinov, M., Belokopytova, P., Taskina, A., Liang, J., Reijnders, M.J.M.F., *et al.* (2022) Anopheles mosquitoes reveal new principles of 3D genome organization in insects. *Nature Communications*, **13**, 1960.
Mahajan, S., Wei, K.H.-C., Nalley, M.J., Gibilisco, L. & Bachtrog, D. (2018) De novo assembly of a young Drosophila Y chromosome using single-molecule sequencing and chromatin conformation capture. *PLOS Biology*, **16**, e2006348.
Mai, D., Nalley, M.J. & Bachtrog, D. (2020) Patterns of Genomic Differentiation in the Drosophila nasuta Species Complex. *Molecular Biology and Evolution*, **37**, 208–220.
Makanga, B.K., Ayala, D., Rahola, N., Bouafou, L.B.A., Johnson, H.F., Heaton, H., *et al.* (2024) A chromosomal reference genome sequence for the malaria mosquito, Anopheles marshallii, Theobald, 1903. *Wellcome Open Research*, **9**, 554.
Massey, J.H., Rice, G.R., Firdaus, A.S., Chen, C., Yeh, S., Stern, D.L., *et al.* (2020) Co-evolving wing spots and mating displays are genetically separable traits in *Drosophila*. *Evolution*, **74**, 1098–1111.
McCulloch, J., Crowley, L.M., University of Oxford and Wytham Woods Genome Acquisition Lab, Darwin Tree of Life Barcoding collective, Wellcome Sanger Institute Tree of Life Management, Samples and Laboratory team, Wellcome Sanger Institute Scientific Operations: Sequencing Operations, *et al.* (2025a) The genome sequence of a lauxanid fly, Meiosimyza decipiens (Loew, 1847). *Wellcome Open Research*, **10**, 245.
McCulloch, J., Crowley, L.M., University of Oxford and Wytham Woods Genome Acquisition Lab, Darwin Tree of Life Barcoding collective, Wellcome Sanger Institute Tree of Life Management, Samples and Laboratory team, Wellcome Sanger Institute Scientific Operations: Sequencing Operations, *et al.* (2025b) The genome sequence of the common stiletto fly, Thereva nobilitata (Fabricius, 1775). *Wellcome Open Research*, **10**, 183.
McCulloch, J., University of Oxford and Wytham Woods Genome Acquisition Lab, Darwin Tree of Life Barcoding collective, Wellcome Sanger Institute Tree of Life programme, Wellcome Sanger Institute Scientific Operations: DNA Pipelines collective, Tree of Life Core Informatics collective, *et al.* (2023) The genome sequence of the yellow-legged black legionnaire, Beris morrisii (Dale, 1841). *Wellcome Open Research*, **8**, 527.
Meisel, R.P., Freeman, J.C., Asgari, D., Llaca, V., Fengler, K.A., Mann, D., *et al.* (2023) New insights into immune genes and other expanded gene families of the house fly, *Musca domestica* , from an improved whole genome sequence. *Archives of Insect Biochemistry and Physiology*, **114**, e22049.

Mitchell, R., Falk, S., Thomas, S., Sivell, O., Sivell, D., Natural History Museum Genome Acquisition Lab, *et al.* (2023a) The genome sequence of a conopid fly, Thecophora atra (Fabricius, 1775). *Wellcome Open Research*, **8**, 358.
Mitchell, R., Natural History Museum Genome Acquisition Lab, Darwin Tree of Life Barcoding collective, Wellcome Sanger Institute Tree of Life Management, Samples and Laboratory team, Wellcome Sanger Institute Scientific Operations: Sequencing Operations, Wellcome Sanger Institute Tree of Life Core Informatics team, *et al.* (2024a) The genome sequence of the Downland Robberfly, Machimus rusticus (Meigen, 1820). *Wellcome Open Research*, **9**, 474.
Mitchell, R., Natural History Museum Genome Acquisition Lab, Darwin Tree of Life Barcoding collective, Wellcome Sanger Institute Tree of Life Management, Samples and Laboratory team, Wellcome Sanger Institute Scientific Operations: Sequencing Operations, Wellcome Sanger Institute Tree of Life Core Informatics team, *et al.* (2024b) The genome sequence of the Brown Heath Robberfly, Tolmerus cingulatus (Fabricius, 1781). *Wellcome Open Research*, **9**, 705.
Mitchell, R., Sivell, O., Natural History Museum Genome Acquisition Lab, Darwin Tree of Life Barcoding collective, Wellcome Sanger Institute Tree of Life Management, Samples and Laboratory team, Wellcome Sanger Institute Scientific Operations: Sequencing Operations, *et al.* (2024c) The genome sequence of a woodlouse fly, Melanophora roralis (Linnaeus, 1758). *Wellcome Open Research*, **9**, 637.
Mitchell, R., Sivell, O., Natural History Museum Genome Acquisition Lab, Darwin Tree of Life Barcoding collective, Wellcome Sanger Institute Tree of Life programme, Wellcome Sanger Institute Scientific Operations: DNA Pipelines collective, *et al.* (2023b) The genome sequence of the Locust Fly, Stomorhina lunata (Fabricius, 1805). *Wellcome Open Research*, **8**, 330.
Mitchell, R., Sivell, O., Sivell, D., Sudworth, J., University of Oxford and Wytham Woods Genome Acquisition Lab, Natural History Museum Genome Acquisition Lab, *et al.* (2024d) The genome sequence of the semaphore fly, Poecilobothrus nobilitatus (Linnaeus, 1767). *Wellcome Open Research*, **9**, 91.
Nash, W.J., Falk, S., Natural History Museum Genome Acquisition Lab, University of Oxford and Wytham Woods Genome Acquisition Lab, Darwin Tree of Life Barcoding collective, Wellcome Sanger Institute Tree of Life Management, Samples and Laboratory team, *et al.* (2024a) The genome sequence of a tachinid fly, Phania funesta (Meigen, 1824). *Wellcome Open Research*, **9**, 713.
Nash, W.J., Halstead, A., Natural History Museum Genome Acquisition Lab, Darwin Tree of Life Barcoding collective, Wellcome Sanger Institute Tree of Life Management, Samples and Laboratory team, Wellcome Sanger Institute Scientific Operations: Sequencing Operations, *et al.* (2024b) The genome sequence of the Golden-tabbed robberfly, Eutolmus rufibarbis (Meigen, 1820). *Wellcome Open Research*, **9**, 593.
Nsango, S.E., Ayala, D., Agbor, J.-P., Johnson, H.F., Heaton, H., Wagah, M.G., *et al.* (2024) A chromosomal reference genome sequence for the malaria mosquito, Anopheles nili, Theobald, 1904. *Wellcome Open Research*, **9**, 599.
Nsango, S.N., Agbor, J.-P., Ayala, D., Johnson, H.F., Heaton, H., Wagah, M.G., *et al.* (2023) A chromosomal reference genome sequence for the malaria mosquito, Anopheles moucheti, Evans, 1925. *Wellcome Open Research*, **8**, 507.
Rahola, N., Ayala, D., Bouafou, L.B.A., Makanga, B.K., Johnson, H.F., Heaton, H., *et al.* (2024) A chromosomal reference genome sequence for the malaria mosquito, Anopheles maculipalpis, Giles, 1902. *Wellcome Open Research*, **9**, 553.
Reinhardt, J.A., Baker, R.H., Zimin, A.V., Ladias, C., Paczolt, K.A., Werren, J.H., *et al.* (2023) Impacts of Sex Ratio Meiotic Drive on Genome Structure and Function in a Stalk-Eyed Fly. *Genome Biology and Evolution*, **15**, evad118.
Renschler, G., Richard, G., Valsecchi, C.I.K., Toscano, S., Arrigoni, L., Ramírez, F., *et al.* (2019) Hi-C guided assemblies reveal conserved regulatory topologies on X and autosomes despite extensive genome shuffling. *Genes & Development*, **33**, 1591–1612.
Richards, S., Liu, Y., Bettencourt, B.R., Hradecky, P., Letovsky, S., Nielsen, R., *et al.* (2005) Comparative genome sequencing of *Drosophila pseudoobscura* : Chromosomal, gene, and *cis* -element evolution. *Genome Research*, **15**, 1–18.
Romine, M.G., Knutie, S.A., Crow, C.M., Vaziri, G.J., Chaves, J.A., Koop, J.A.H., *et al.* (2022) The genome sequence of the avian vampire fly (*Philornis downsi*), an invasive nest parasite of Darwin's finches in Galápagos. *G3 Genes|Genomes|Genetics*, **12**, jkab414.
Sims, I., Raper, C., Sivell, O., Natural History Museum Genome Acquisition Lab, Darwin Tree of Life Barcoding collective, Wellcome Sanger Institute Tree of Life Management, Samples and Laboratory team, *et al.* (2025) The genome sequence of the Stable Fly, Stomoxys calcitrans (Linnaeus, 1758). *Wellcome Open Research*, **10**, 48.
Sivell, D., Sivell, O., Natural History Museum Genome Acquisition Lab, Darwin Tree of Life Barcoding collective, Wellcome Sanger Institute Tree of Life programme, Wellcome Sanger Institute Scientific Operations: DNA Pipelines collective, *et al.* (2023a) The genome sequence of the Vagrant Hoverfly,

Eupeodes corollae (Fabricius, 1794). *Wellcome Open Research*, **8**, 112.
Sivell, O., McAlister, E., Mitchell, R., Falk, S., Natural History Museum Genome Acquisition Lab, University of Oxford and Wytham Woods Genome Acquisition Lab, *et al.* (2024a) The genome sequence of a soldierfly, Chorisops tibialis (Meigen, 1820). *Wellcome Open Research*, **9**, 715.
Sivell, O., Mitchell, R., Sivell, D., Natural History Museum Genome Acquisition Lab, Darwin Tree of Life Barcoding collective, Wellcome Sanger Institute Tree of Life programme, *et al.* (2023b) The genome sequence of a snail-killing fly, Pherbina coryleti (Scopoli, 1763). *Wellcome Open Research*, **8**, 30.
Sivell, O., Mitchell, R., Sivell, D., Natural History Museum Genome Acquisition Lab, Darwin Tree of Life Barcoding collective, Wellcome Sanger Institute Tree of Life programme, *et al.* (2023c) The genome sequence of a soldierfly, Nemotelus nigrinus (Fallén, 1817). *Wellcome Open Research*, **8**, 240.
Sivell, O., Natural History Museum Genome Acquisition Lab, Darwin Tree of Life Barcoding collective, Wellcome Sanger Institute Tree of Life programme, Wellcome Sanger Institute Scientific Operations: DNA Pipelines collective, Tree of Life Core Informatics collective, *et al.* (2023d) The genome sequence of a bluebottle, Calliphora vomitoria (Linnaeus, 1758). *Wellcome Open Research*, **8**, 93.
Sivell, O., Raper, C., Mitchell, R., Sivell, D., Natural History Museum Genome Acquisition Lab, Darwin Tree of Life Barcoding collective, *et al.* (2024b) The genome sequence of a robberfly, Dysmachus trigonus (Meigen, 1804). *Wellcome Open Research*, **9**, 453.
Sivell, O., Sivell, D., Mitchell, R., Webb, J.A., Falk, S., Natural History Museum Genome Acquisition Lab, *et al.* (2025) The genome sequence of a snipefly, Rhagio lineola Fabricius, 1794. *Wellcome Open Research*, **10**, 154.
Sivell, O., Sivell, D., Natural History Museum Genome Acquisition Lab, Darwin Tree of Life Barcoding collective, Wellcome Sanger Institute Tree of Life programme, Wellcome Sanger Institute Scientific Operations: DNA Pipelines collective, *et al.* (2021a) The genome sequence of the St Mark's fly, Bibio marci (Linnaeus, 1758). *Wellcome Open Research*, **6**, 285.
Sivell, O., Sivell, D., Natural History Museum Genome Acquisition Lab, Darwin Tree of Life Barcoding collective, Wellcome Sanger Institute Tree of Life programme, Wellcome Sanger Institute Scientific Operations: DNA Pipelines collective, *et al.* (2021b) The genome sequence of a snail-killing fly, Coremacera marginata (Fabricius, 1775). *Wellcome Open Research*, **6**, 338.
Sivell, O., Sivell, D., Natural History Museum Genome Acquisition Lab, Darwin Tree of Life Barcoding collective, Wellcome Sanger Institute Tree of Life programme, Wellcome Sanger Institute Scientific Operations: DNA Pipelines collective, *et al.* (2022) The genome sequence of a hoverfly, Xanthogramma pedissequum (Harris, 1776). *Wellcome Open Research*, **7**, 38.
Sivell, O., Sivell, D., Natural History Museum Genome Acquisition Lab, Darwin Tree of Life Barcoding collective, Wellcome Sanger Institute Tree of Life programme, Wellcome Sanger Institute Scientific Operations: DNA Pipelines collective, *et al.* (2023e) The genome sequence of a Tiger Cranefly, Nephrotoma flavescens (Linnaeus, 1758). *Wellcome Open Research*, **8**, 148.
Sivell, O., Sivell, D., Webb, J.A., Mitchell, R., Ashworth, M., Natural History Museum Genome Acquisition Lab, *et al.* (2024c) The genome sequence of the acalyptrate fly Dryomyza anilis Fallén, 1820. *Wellcome Open Research*, **9**, 440.
Sivell, O., Webb, J., Mitchell, R., Sivell, D., Natural History Museum Genome Acquisition Lab, Darwin Tree of Life Barcoding collective, *et al.* (2024d) The genome sequence of a fold-wing cranefly, Ptychoptera albimana (Fabricius, 1787). *Wellcome Open Research*, **9**, 598.
Small, S.T., Labbé, F., Lobo, N.F., Koekemoer, L.L., Sikaala, C.H., Neafsey, D.E., *et al.* (2020) Radiation with reticulation marks the origin of a major malaria vector. *Proceedings of the National Academy of Sciences*, **117**, 31583–31590.
Smith, M., Natural History Museum Genome Acquisition Lab, Darwin Tree of Life Barcoding collective, Wellcome Sanger Institute Tree of Life programme, Wellcome Sanger Institute Scientific Operations: DNA Pipelines collective, Tree of Life Core Informatics collective, *et al.* (2022) The genome sequence of Gymnosoma rotundatum (Linnaeus, 1758), a parasitoid ladybird fly. *Wellcome Open Research*, **7**, 104.
Srinivasan, S., Ghosh, C., Das, S., Thakare, A., Singh, S., Ganesh, A., *et al.* (2022) Identification of a TNF-TNFR-like system in malaria vectors (Anopheles stephensi) likely to influence Plasmodium resistance. *Scientific Reports*, **12**, 19079.
Tandonnet, S., Krsticevic, F., Basika, T., Papathanos, P.A., Torres, T.T. & Scott, M.J. (2023) A chromosomal-scale reference genome of the New World Screwworm, *Cochliomyia hominivorax*. *DNA Research*, **30**, dsac042.
Taylor, H.R., Ritchie-Parker, H., Wellcome Sanger Institute Tree of Life programme, Wellcome Sanger Institute Scientific Operations: DNA Pipelines collective, Tree of Life Core Informatics collective, & Darwin Tree of Life Consortium. (2025) The genome sequence of the pine hoverfly, Blera fallax (Linnaeus, 1758). *Wellcome Open Research*, **8**, 89.
Taylor, S.C., Crowley, L.M., Luker, S., Harvey, M., Natural History Museum Genome Acquisition Lab,

University of Oxford and Wytham Woods Genome Acquisition Lab, *et al.* (2023) The genome sequence of the Downland Villa bee-fly, Villa cingulata (Meigen, 1804). *Wellcome Open Research*, **8**, 526.
Thomas, S., Mitchell, R., Crowley, L.M., Natural History Museum Genome Acquisition Lab, University of Oxford and Wytham Woods Genome Acquisition Lab, Darwin Tree of Life Barcoding collective, *et al.* (2023) The genome sequence of the Kite-tailed Robberfly, Machimus atricapillus (Fallén, 1814). *Wellcome Open Research*, **8**, 113.
Torosin, N.S., Anand, A., Golla, T.R., Cao, W. & Ellison, C.E. (2020) 3D genome evolution and reorganization in the Drosophila melanogaster species group. *PLOS Genetics*, **16**, e1009229.
Trinca, V., Carli, S., Uliana, J.V.C., Garbelotti, C.V., Mendes Da Silva, M., Kunes, V., *et al.* (2023) Biocatalytic potential of Pseudolycoriella CAZymes (Sciaroidea, Diptera) in degrading plant and fungal cell wall polysaccharides. *iScience*, **26**, 106449.
University of Oxford and Wytham Woods Genome Acquisition Lab, Darwin Tree of Life Barcoding collective, Wellcome Sanger Institute Tree of Life programme, Wellcome Sanger Institute Scientific Operations: DNA Pipelines collective, Tree of Life Core Informatics collective, Nash, W., *et al.* (2022a) The genome sequence of the ferruginous bee-grabber, Sicus ferrugineus (Linnaeus, 1761). *Wellcome Open Research*, **7**, 91.
University of Oxford and Wytham Woods Genome Acquisition Lab, Darwin Tree of Life Barcoding collective, Wellcome Sanger Institute Tree of Life programme, Wellcome Sanger Institute Scientific Operations: DNA Pipelines collective, Tree of Life Core Informatics collective, Nash, W., *et al.* (2022b) The genome sequence of Tachina fera Linnaeus, 1761, a tachinid fly. *Wellcome Open Research*, **7**, 103.
Urban, J.M., Foulk, M.S., Bliss, J.E., Coleman, C.M., Lu, N., Mazloom, R., *et al.* (2021) High contiguity de novo genome assembly and DNA modification analyses for the fungus fly, Sciara coprophila, using single-molecule sequencing. *BMC Genomics*, **22**, 643.
Wawman, D.C., Insley, H., University of Oxford and Wytham Woods Genome Acquisition Lab, Darwin Tree of Life Barcoding collective, Wellcome Sanger Institute Tree of Life Management, Samples and Laboratory team, Wellcome Sanger Institute Scientific Operations: Sequencing Operations, *et al.* (2025a) The genome sequence of the Grouse Louse Fly, Ornithomya chloropus (Bergroth, 1901). *Wellcome Open Research*, **10**, 191.
Wawman, D.C., University of Oxford and Wytham Woods Genome Acquisition Lab, Darwin Tree of Life Barcoding collective, Wellcome Sanger Institute Tree of Life Management, Samples and Laboratory team, Wellcome Sanger Institute Scientific Operations: Sequencing Operations, Wellcome Sanger Institute Tree of Life Core Informatics team, *et al.* (2025b) The genome sequence of the Finch Louse Fly, Ornithomya fringillina (Curtis, 1836). *Wellcome Open Research*, **10**, 185.
Wei, K.H.-C., Mai, D., Chatla, K. & Bachtrog, D. (2022) Dynamics and Impacts of Transposable Element Proliferation in the *Drosophila nasuta* Species Group Radiation. *Molecular Biology and Evolution*, **39**, msac080.
Wolton, R., Drake, M., Natural History Museum Genome Acquisition Lab, Darwin Tree of Life Barcoding collective, Wellcome Sanger Institute Tree of Life Management, Samples and Laboratory team, Wellcome Sanger Institute Scientific Operations: Sequencing Operations, *et al.* (2025) The genome sequence of the Ornate Brigadier fly, Odontomyia ornata (Meigen, 1822). *Wellcome Open Research*, **10**, 272.
Zamyatin, A., Avdeyev, P., Liang, J., Sharma, A., Chen, C., Lukyanchikova, V., *et al.* (2021) Chromosome-level genome assemblies of the malaria vectors *Anopheles coluzzii* and *Anopheles arabiensis*. *GigaScience*, **10**, giab017.
